# Comparison of videolaryngoscopy and direct laryngoscopy by German paramedics during out-of-hospital cardiopulmonary resuscitation; an observational prospective study

**DOI:** 10.1186/s12873-020-00316-z

**Published:** 2020-03-23

**Authors:** Joachim Risse, Christian Volberg, Thomas Kratz, Birgit Plöger, Andreas Jerrentrup, Dirk Pabst, Clemens Kill

**Affiliations:** 1 Center of Emergency Medicine, University Hospital Essen, Hufelandstrasse 55, 45122 Essen, Germany; 2 Department of Anesthesiology and Intensive Care Medicine, Philipps-University Marburg, 35033 Marburg, Germany; 3 Department of Anesthesia and Intensive Care Medicine, 21240 Talant, Côte-d’Or, Dijon, France; 4 Department of Emergency Medicine, Philipps-University Marburg, 35033 Marburg, Germany

**Keywords:** Cardiopulmonary resuscitation, Endotracheal intubation, Paramedics, Out-of-hospital cardiac arrest, Videolaryngoscopy

## Abstract

**Background:**

Videolaryngoscopy (VL) has become a popular method of intubation (ETI). Although VL may facilitate ETI in less-experienced rescuers there are limited data available concerning ETI performed by paramedics during CPR. The goal was to evaluate the impact VL compared with DL on intubation success and glottic view during CPR performed by German paramedics. We investigated in an observational prospective study the superiority of VL by paramedics during CPR compared with direct laryngoscopy (DL).

**Methods:**

In a single Emergency Medical Service (EMS) in Germany with in total 32 ambulances paramedics underwent an initial instruction from in endotracheal intubation (ETI) with GlideScope® (GVL) during resuscitation. The primary endpoint was good visibility of the glottis (Cormack-Lehane grading 1/2), and the secondary endpoint was successful intubation comparing GVL and DL.

**Results:**

In total *n* = 97 patients were included, *n* = 69 with DL (*n* = 85 intubation attempts) and *n* = 28 VL (*n* = 37 intubation attempts). Videolaryngoscopy resulted in a significantly improved visualization of the larynx compared with DL. In the group using GVL, 82% rated visualization of the glottis as CL 1&2 versus 55% in the DL group (*p* = 0.02). Despite better visualization of the larynx, there was no statistically significant difference in successful ETI between GVL and DL (GVL 75% vs. DL 68.1%, *p* = 0.63).

**Conclusions:**

We found no difference in Overall and First Pass Success (FPS) between GVL and DL during CPR by German paramedics despite better glottic visualization with GVL. Therefore, we conclude that education in VL should also focus on insertion of the endotracheal tube, considering the different procedures of GVL.

**Trial registration:**

German Clinical Trial Register DRKS00020976, 27. February 2020 retrospectively registered.

## Background

Maintaining an open airway is one of the most important procedures in emergency care during advanced life support (ALS) resuscitation and is essential for adequate ventilation of the patient. Emergency medical services (EMS) in Germany is designed as a two-tiered system including a physician-staffed EMS unit in all life-threatening cases and an ALS-Ambulance with paramedics. As soon as the emergency physician is on scene, procedures such as endotracheal Intubation (ETI) are performed by the physician. Due to the higher availability of paramedic-staffed ambulances, in many cases the paramedics are on scene before the arrival of the EMS physician. Although paramedics are trained in ALS including direct laryngoscopy and endotracheal intubation, the rate of performing endotracheal intubation by paramedics before the arrival of the emergency physician unit is low. Therefore we investigated the effect of VL compared to DL by paramedics during CPR before arrival of the emergency physician on both visibility of the glottis and intubation success rate.

Adequate ventilation, improved oxygenation, and avoidance of aspiration are important factors concerning the rate of ROSC as well as the neurological outcome of a patient undergoing CPR [1, 2]. Current updated international recommendations for advanced airway management from the International Liaison Committee on Resuscitation (ILCOR) suggest supraglottic devices for adults with Out-of-hospital cardiac arrest (OHCA) in settings with a low intubation success rate [3]. In case of less experienced providers they recommended mask ventilation or supraglottic devices in order to not interrupt chest compressions.

In the Anglo-American paramedic system, the success rates for prehospital ETI using DL are between 71 and 75% [4, 5]. In contrast to Anglo-American paramedic system to For German paramedics, ETI during CPR is generally a rare event as the attending physician usually carries out the procedure. Some investigators have shown that untrained users have a 51% rate of successful intubation with DL (MacIntosh) [6]. In contrast, with VL inexperienced users have an especially steep learning curve and a significantly higher success rate [6, 7]. Videolaryngoscopy is superior to DL when the first attempt at intubation has failed and is associated with a reduction in esophageal intubations [8, 9].

Current studies could demonstrate that VL improves glottic opening but do not improve First Pass Success (FPS) [10–12]. On the other Hand there is valid data that VL improves FPS [13–15]. Moreover, data exist showing that the overall success rate of ETI by inexperienced physicians during CPR is significantly higher with VL than with DL [4, 9, 16]. During CPR with ongoing chest compression using VL for ETI might result in reduced interruption of chest compressions [17–19]. Several studies have shown that in pre-hospital settings there is an alarmingly high rate of failed ETI, especially when performed by non-physicians [20–22].

We investigated the impact of using videolaryngoscopy (VL) instead of direct laryngoscopy (DL) by paramedics in out-of-hospital cardiac arrest before arrival of the emergency physician on scene in a semi-rural county in Germany. We focused our investigation on glottic opening and overall und First Pass success (FPS).

## Methods

### Study design and time period

With approval by the institutional ethics committee, we designed a prospective observational study comparing DL and GVL by paramedics in out-of-hospital cardiac arrest (OHCA) without an emergency physician on scene. We performed our investigation under actual field conditions over a period of 4 years to include a sufficient number of cases. ETI should be performed either with VL (GlideScope® Ranger, GVL) or the standard DL (MacIntosh) depending on the availability of VL at time of CPR. Therefore four GVL devices were allocated on four of the 32 ambulances for six-month to assure experience and rotated after 6 month to the next four ambulances of the EMS agency. The rotation of the 4 GVL continued until the end of the investigation period of 4 years.

### Study setting and population

The inclusion criteria were patients in non-traumatic cardiac arrest, ongoing basic life support (BLS) with chest compressions and bag-mask-ventilation, and absence of an emergency physician on scene.

The exclusion criteria were patients aged less than 12 years, the presence of an emergency physician on scene, and the primary use of a supraglottic airway device by the paramedics.

All paramedics of EMS received a training course explaining handling of GVL before starting the study. For this purpose, a manikin exercise phantom head was used for ETI training, and the correct and different handling of the GVL was practiced under medical supervision, ca. 20 intubations (JR, TK, CK). A new standard operating procedure (SOP) “Airway management with videolaryngoscopy (GVL)” was developed before commencing the study and was implemented in the annual paramedic training. The content of the SOP was: If the ambulance was equipped with GVL it was mandatory using first line GVL for securing the airway during ALS procedure without EMS physician on scene. The new SOP and an instruction manual, with instructions for the different technique and preparation of the tube with a rigid stylet for hyper angulated blades, were made available to all paramedics via the company’s intranet. After Training period paramedics had the opportunity to practice on a manikin with medical supervision during the 6 month of availability of the GVL device at their EMS base. There was no additional training in patients as in the operation theatre.

Our primary endpoint was the visibility of the glottis with a Cormack-Lehane (CL) score of 1 or 2, and the secondary endpoint was the overall ETI success and the First Pass Success FPS rate during out-of-hospital CPR.

An ETI attempt was defined whenever a VL or DL blade passed the teeth to intubate and secure the airway during advanced cardiac life support (ACLS). The number of required attempts was recorded. Successful ETI was defined as the successful placement of an endotracheal tube and correct pulmonary ventilation with a positive capnography. The Success with a positive EtCO2 waveform was confirmed by the EMS physician arriving on scene. A maximum of two attempts were allowed per the internal ALS protocol of the EMS. If ETI failed, paramedics were recommended to use a laryngeal mask to secure the airway. After every ETI attempt during CPR by paramedics, the research team sent a self-report questionnaire to the paramedic team to acquire data.

### Outcome measures and data analysis

Non-normally distributed variables were expressed as the median and upper and lower quartiles (Q.25 and Q.75). These data were analyzed by using the Mann–Whitney U test. Data were also presented as percentages with confidence intervals (CI). Differences in frequency were tested for significance using the chi-square test and for small cell occupations using Fisher’s exact test. To test for correlations, the point biserial correlation coefficient (r.pb) was used. The significance level was set to alpha = 0.05. Data are presented as histograms and box-and-whisker diagrams. All statistical calculations were performed using the statistics packages SPSS (version 22) and BiAS for Windows (version 11.03, Epsilon Verlag, 2016).

## Results

**Fig. 1:**
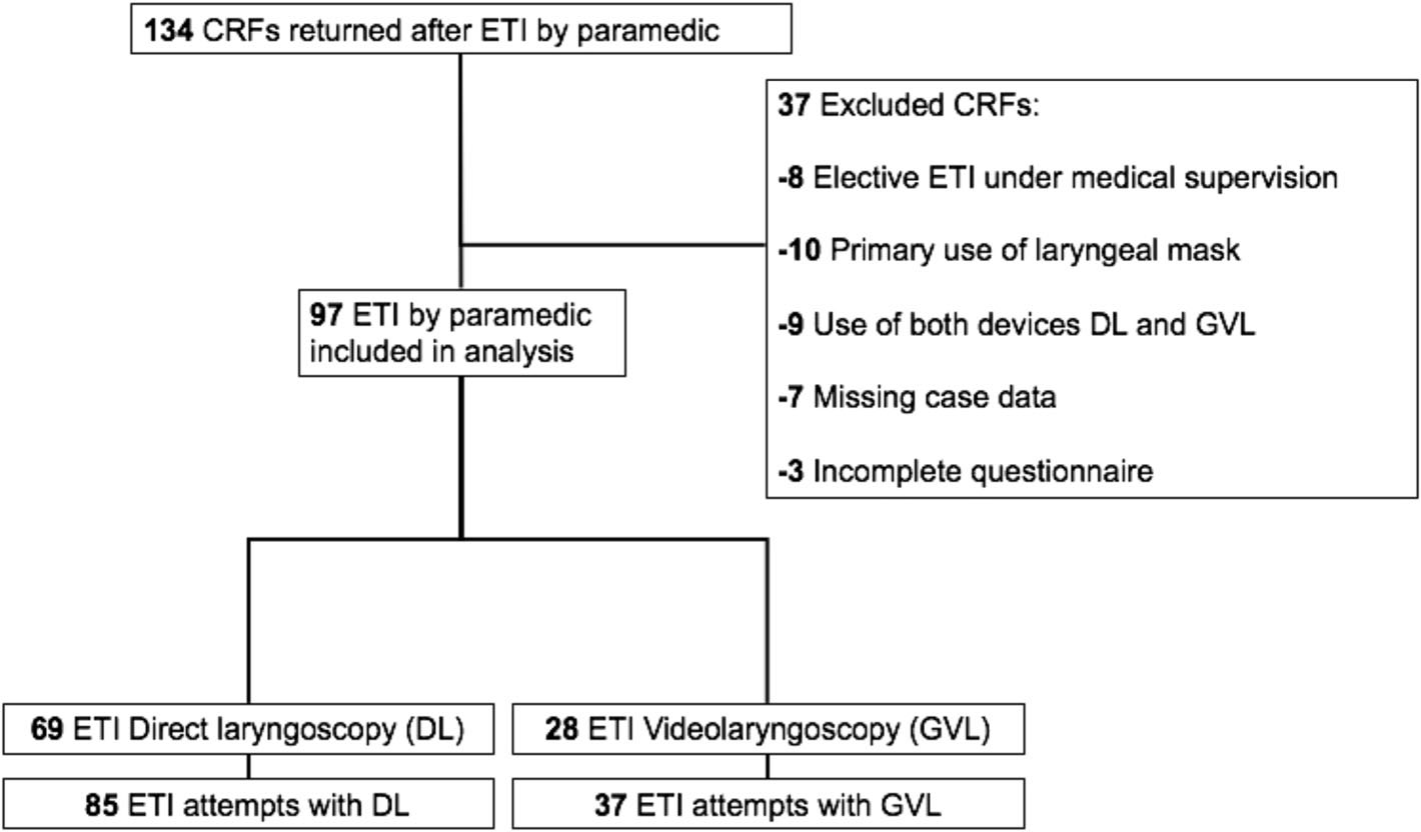
Diagram data evaluation

**Fig. 2:**
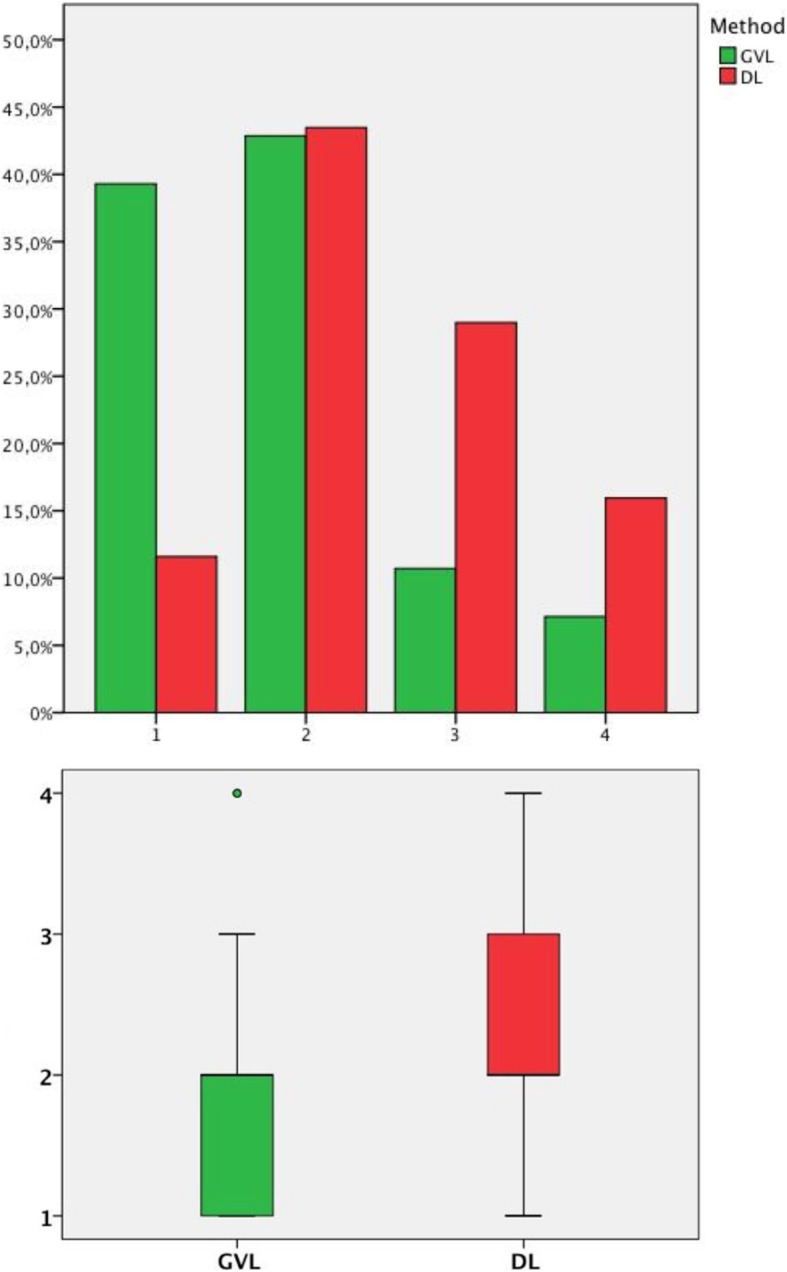
Frequency distribution of the Cormack and Lehane grading (CL) between videolaryngoscopy (GVL) and direct laryngoscopy (DL). 1. bar graph x-axis CL Grade, y-axis number in percent, method GVL (green) and DL (red). 2. boxplot x-axis method GVL (green) and DL (red), y-axis CL Grade 1–4

In total 134 case report forms (CRFs) from patients after CPR were collected. For various reasons, 37 CRF were excluded from further evaluation (see Fig. 1). Finally, the CRF’s of *n* = 97 patients were included, *n* = 69 with use of DL (with *n* = 85 intubation attempts) and *n* = 28 with use of GVL (with *n* = 37 intubation attempts). Paramedics’ professional experience in years (7 yrs. in DL vs 6.5 yrs. in GVL, *p* = 0.48) and the estimated number of conventional (DL) intubations (*n* = 30 DL vs *n* = 22.5 GVL, *p* = 0.14) performed previously by the paramedics were similar in both groups. For the CL grade 1–4, a statistically significant difference in our data could be shown between the two groups (*p* = 0.002) (see Fig. 2). With GVL a CL grade of 1 was significantly more frequent, with a difference of 27.7% compared with DL (*p* = 0.004) (see Table 1). The proportion of dichotomized CL grades 1&2 vs 3&4 were statistically significantly different in the GVL group compared with the DL group. CL grades 1&2 represent an easier ETI in contrast to Grades 3&4, which represent a more difficult ETI. CL grade 1&2 was statistically significantly more frequent in the GVL group than in the DL group (*p* = 0,02) (see Table 1). Regardless of the method used for an ETI by paramedics, our data showed that the number of unsuccessful ETIs increased with a higher CL grade (r.pb = 0.614, *p* < 0.0001) (see Table 2).

**Table 1:** Comparison view of the larynx by Cormack-Lehane classification system video–laryngoscopy (GVL) vs. direct laryngoscopy (DL)

***CL-Grade***	***GVL***	***DL***	***p-value***
***I***	***11 (39,29%)***	***8 (11,59%)***	***0,004***
***II***	***12 (42,86%)***	***30 (43,48%)***	***1***
***III***	***3 (10,71%)***	***20 (28,99%)***	***0,07***
***IV***	***2 (7,14%)***	***11 (15,94%)***	***0,34***
**Dichotomized CL-Grade**	**GVL**	**DL**	***p-value***
I + II (easier ETI)	23 (82,15%)	38 (55,07%)	**0,02**
III + IV (difficult ETI)	5 (17,85%)	31 (44,93%)	**0,02**

**Table 2:** Comparison total endotracheal intubation (ETI) success depending on the Cormack-Lehane classification system

CL-Grade	**I**	**II**	**III**	**IV**
***Total no. ETI successful***	17 (89,5%)	37 (88,1%)	12 (52,2%)	2 (15,4%)
***Total no. ETI unsuccessful***	2 (10,5%)	5 (11,9%)	11 (47,8%)	11 (84,6%)

point biserial correlation coefficient r.pb = 0,614, *p* < 0,0001

The other focus of the investigation (overall ETI success and First Pass success FPS) did not differ significantly between the GVL group and the DL group (*p* = 0,63) (see Table 3). There was no statistically significant difference between the two groups in the number of intubation attempts (1 or 2), (*p* = 0.4) (see Table 4). Also, the success rate on the first and second attempt was similar in both groups. The difference found was not significant (*p* = 0.2) (see Table 5).

**Table 3:** Comparison of overall successful Endotracheal Intubation (ETI)

	ETI successful	ETI unsuccessful	
**GVL**	21 (75%)	7 (25%)	28
**DL**	47 (68.1%)	22 (31.9%)	69
	68	29	97
	Fisher’s exact test two-tailed *P*-value is 0.626777

**Table 4:** Comparison of the number of Endotracheal Intubation (ETI) attempts

	One ETI attempt	Two ETI attempts	
**GVL**	19 (67.9%)	9 (32.1%)	28
**DL**	53 (76.8%)	16 (23.2%)	69
	72	25	97
	Fisher’s exact test two-tailed *P*-value is 0.4434

**Table 5:** Comparison of the success rate on the 1st and 2nd attempt

	First attempt ETI success	Second attempt ETI success	
**GVL**	15 (71.4%)	6 (28.6%)	21
**DL**	40 (85.1%)	7 (14.9%)	47
	55	13	68
	Fisher’s exact test two-tailed *P*-value is 0.199879		

## Discussion

Paramedics with limited experience in both DL and videolaryngoscopic ETI might have improved success rates using GVL as a first-line device in emergency airway management with CPR. Our results from this out-of-hospital observational study demonstrate improved visualization of the larynx with GVL. Despite better visualization of the larynx with GVL, the first pass success FPS and the overall success rates for ETI were not improved compared with DL during CPR when performed by German paramedics. This may be due to less experience in handling a videolaryngoscope and infrequent opportunities for German paramedics to perform ETI in general.

Previous investigations showed a significantly higher intubation success rate by inexperienced users during CPR with GVL than with DL [16, 19]. Lee et al. investigated tracheal intubation during in-hospital cardiopulmonary resuscitation [16]. These results from clinical research cannot simply be transferred to the out of hospital setting. However we were not able to show an increased success rate for ETI when performed by German paramedics who are less experienced in the procedure.

Endotracheal intubation during resuscitation is frequently associated with a difficult airway and shows FPS with VL, depending on the study, between 73 and 94%, even for experienced physicians [16, 17]. Most of the previous studies observing GVL during CPR investigated experienced physicians or were just simulation studies with mannequins [17, 23–25]. The differences to our results might be based on user experience (physicians, non-physicians) with the procedure. We suspect a broad range of user experiences across individuals and studies.

Ducharme et al. saw similar relevant results in their investigation of American paramedics over a period of 34 months. The group showed that VL had similar FPS rates and even better laryngoscopic visualization compared with DL. They used the King Vision® videolaryngoscope, whereas our investigation used the GlideScope® Ranger [26]. In addition, our study results showed a trend towards a higher rate of successful ETI on the second attempt with GVL. This might be based on an immediate learning process from the first attempt to the second attempt with VL. A minimal optimization during the second attempt (blood and secretion suction, cleaning the lens, view of the monitor) might be enough in such a situation to successfully intubate with GVL. Nouruzi-Sedeh et al. showed a success rate of more than 90% on the first attempt in their investigation with personnel untrained in intubation using GVL. In the second attempt, all subjects were successfully intubated with the GlideScope technique [6]. In this context, due to the small number of cases, we could only see a statistically insignificant trend in our data towards a higher rate of successful ETI on the second attempt with GVL during CPR.

During out-of-hospital CPR there are multiple external influences and stressors on the paramedic team, for example, the unfamiliar environment, lighting etc. Russo et al. postulate that videolaryngoscopes are helpful for emergency intubations, but sufficient experience in dealing with the devices is essential. They also showed the limitations of videolaryngoscopes, e.g. blood, vomit or secretions in front of the lens, as well as bright light producing glare on the screen [27]. These stressors might also be responsible for the poor performance observed with both devices.

### Limitations

The first limitation of our study is related to its design. We performed a preliminary observation trial with paramedics from single EMS area. For that reason, our study sample was small and unbalanced. For paramedics in Germany, ETI is a rare event, and we performed our investigation under actual field conditions over a period of 4 years to include a sufficient number of cases. In most cases of pre-hospital emergency medicine in Germany, an emergency physician performs intubation. To obtain a larger case number in an adequate investigation time period, several different EMS should be included in further investigations. All paramedics were instructed to report during the investigation period. There was no cross-checking how many patients underwent ETI by paramedics without a corresponding CRF returned. In addition, there is a possibility of reporting bias. Despite anonymization of the questionnaires and optional participation for the paramedics in this investigation, positive results and positive occurrences might be reported more often than negative ones. The instruction for the paramedics in using GVL instead of DL was only a manikin training without additional training in patients in elective surgery, e.g. All paramedics underwent training in DL in their professional education much more intensively than training of GVL for this study, so there might be a bias in favour of DL as a limitation of this study.

A further limitation is due to the different levels of training with DL and GVL of the individual paramedics in the single investigated EMS. Based on the variability of individual intubation experience among paramedics in this single EMS, the results cannot be transferred to another EMS. Furthermore, our study was conducted over 4 years and there has possibly been an increase in SGA use by paramedics, because more recent studies indicated that SGAs could be equivalent or better than ETI [28, 29]. This might have also an effect on the small number of cases in the whole investigation period.

We used the GlideScope® Ranger videolaryngoscope in our investigation, while the group of Ducharme et al. for example used the King Vision® videolaryngoscope [26]. The two studies obtained similar results; however, there are currently many different videolaryngoscopes with varying designs and quality available on the market. For these reasons, our study results should not be generalized, and further investigation is needed.

## Conclusions

We found no difference in Overall and First Pass Success FPS between GVL and DL during CPR by German paramedics despite better glottic visualization with GVL.

Therefore, we conclude that more training in VL for German paramedics is needed and education in VL should focus on insertion of the endotracheal tube, considering the different procedures of GVL.

## Data Availability

The data that support the findings of this study are available from the corresponding author. The datasets used and analyzed during the current study are available from the corresponding author on reasonable request.

## Abbreviations

ACLS: Advanced cardiac life support
ALS: Advanced life support
CL grading: Cormack-Lehane grading
CPR: Cardiopulmonary resuscitation
CRF: Case report form
CK: Clemens Kill
EMS: Emergency medical services
ETI: Endotracheal intubation
DL: Direct laryngoscopy
FPS: First Pass Success
GVL: GlideScope® videolaryngoscopy
ILCOR: International Liaison Committee on Resuscitation
JR: Joachim Risse
OHCA: Out-of-hospital Cardiac Arrest
ROSC: Return of spontaneous circulation
SOP: Standard operating procedure
TK: Thomas Kratz
VL: Videolaryngoscopy
